# In Situ Synthesis of Poly(butyl methacrylate) in Anodic Aluminum Oxide Nanoreactors by Radical Polymerization: A Comparative Kinetics Analysis by Differential Scanning Calorimetry and ^1^H-NMR

**DOI:** 10.3390/polym13040602

**Published:** 2021-02-17

**Authors:** Laia León-Boigues, Luis Andrés Pérez, Carmen Mijangos

**Affiliations:** Instituto de Ciencia y Tecnología de Polímeros, ICTP-CSIC, Juan de la Cierva 3, 28006 Madrid, Spain; laleboi@ictp.csic.es (L.L.-B.); lperez@ictp.csic.es (L.A.P.)

**Keywords:** confined polymerization, butyl methacrylate (BMA), kinetic analysis by ^1^H-NMR spectroscopy, polymer nanostructures

## Abstract

In this work, we explore the ability to generate well-defined poly(butyl methacrylate) (PBMA) nanostructures by “in situ” polymerization of butyl methacrylate monomer (BMA). PBMA nanostructures of high and low aspect ratios have been successfully obtained through the free radical polymerization (FRP) of a BMA monomer in anodic aluminum oxide (AAO) nanoreactors of suitable size. A polymerization kinetics process has been followed by differential scanning calorimetry (DSC) and proton Nuclear Magnetic Resonance spectroscopy (^1^H-NMR).The determination of the kinetics of polymerization through DSC is based on a quick and direct analysis of the exothermic polymerization process, whereas the analysis through ^1^H-NMR also allows the unambiguous chemical analysis of the resulting polymer. When compared to bulk polymerization, both techniques demonstrate confinement effects. Moreover, DSC and ^1^H-NMR analysis give the same kinetics results and show a gel-effect in all the cases. The number average molecular weight (Mn) of the PBMA obtained in AAO of 60–300 nm are between 30·10^3^–175·10^3^ g/mol. Even if the Mn value is lower with respect to that obtained in bulk polymerization, it is high enough to maintain the polymer properties. As determined by SEM morphological characterization, once extracted from the AAO nanoreactor, the polymer nanostructures show controlled homogeneous aspect/size all throughout the length of nanopillar over a surface area of few cm^2^. The Young’s modulus of low aspect ratio PBMA nanopillars determined by AFM gives a value of 3.1 ± 1.1 MPa. In this work, a 100% of PBMA polymer nanostructures are obtained from a BMA monomer in AAO templates through a quick double process: 30 min of monomer immersion at room temperature and 90 min of polymerization reaction at 60 °C. While the same nanostructures are obtained by polymer infiltration of PBMA at 200 °C in about 6 h, polymerization conditions are much softer than those corresponding to the polymer infiltration process. Furthermore, the ^1^H-NMR technique has been consolidated as a tool for studying the kinetics of the copolymerization reactions in confinement and the determination of monomer reactivity ratios.

## 1. Introduction

Nanoporous anodic aluminum oxide (AAO) templates are considered ideal hard geometries to prepare hierarchical one-dimensional polymer morphologies with dimensions on the nanometric scale. Polymer infiltration into AAO nanocavities has been the traditional and most widely used method to prepare polymer morphologies in the nanoscale [1,2,3,4,5,6,7]. This nanomolding method requires high temperature and a relatively long period time, from hours to days. Moreover, through this method, it is possible to study and understand the polymer properties under confinement [8,9,10,11,12,13,14,15].

Very recently, the “in situ” polymerization of a monomer in AAO templates has been presented as an alternative and complementary method to prepare the same polymer morphologies, without the limitations of strong polymer infiltration conditions and, therefore, extending the possibilities of nanostructuring to the entire library of polymers, including polymers that cannot be dissolved or molten. Nevertheless, only few studies have been reported in the literature dealing with polymerization kinetics although the interest in the “in situ” polymerization method has increased in recent years [16,17,18,19,20,21,22]. The studies go from free radical polymerization of different monomers to different polymerization mechanisms.

For example, Giussi et al. [23] studied the reaction kinetics of free radical vinyl polymerization of styrene within AAO nanoreactors, Salsamendi et al. [24] studied the polymerization reaction of a fluorinated acrylic monomer as an example of non-gel effect polymerization mechanism, Sanz et al. [25] studied the polymerization of a methyl methacrylate monomer as an example of gel effect polymerization mechanism and Zhao et al. [26] also reported the reaction kinetics of methyl methacrylate. Tarnacka et al. and Sanz et al. [27,28] reported the step polymerization reaction in AAO templates and very recently Giussi et al. [29] carried out the atom transfer radical polymerization (ATRP) in AAO nanoreactors. As demonstrated in the quoted works, the polymerization of a monomer occurs within the nanocavities of AAO template at relatively low temperatures and short times. Each cavity acts as an isolated reactor, and its dimensions (pore diameter and length) can be easily adjusted.

Related to the butyl methacrylate (BMA) monomer, although there are a few studies on polymerizations in bulk conditions [30,31,32,33] and several on polymerization of acrylic monomers in other confined geometries such as porous glasses [34,35], the “in situ” polymerization reaction in confinement conditions in AAO templates has been scarcely reported in literature [28,36,37]. In particular, the polymerization of butyl methacrylate monomer (BMA) or 2-hydroxyethyl acrylate monomer (HEA) in AAO templates has never been reported.

Regarding the kinetic analysis of “in situ” polymerization in AAO templates, most studies employed differential scanning calorimetry (DSC) to monitor the heat released from the polymerizing sample as a function of time or infrared (IR) spectroscopy to follow the formation of the polymer chemical structure. To the best of our knowledge, none of the studies reported so far have employed proton Nuclear Magnetic Resonance spectroscopy (^1^H-NMR) to analyze the kinetics of “in situ” polymerization in AAO templates, even though this technique is widely employed for the analysis of kinetics in bulk polymerization.

The objective of this work is double, first the fabrication of nanostructured poly(butyl methacrylate) (PBMA) polymers of suitable size and properties by “in situ” polymerization of BMA monomer within AAO nanoreactors and the study of the reaction kinetics by ^1^H-NMR spectroscopy and the comparison with DSC technique. To that aim, AAO nanoreactors of controlled dimensions were fabricated, DSC and ^1^H-NMR techniques were employed to follow the free radical polymerization reaction as a function of time by monitoring the heat released and the monomer/polymer proton signals, respectively. The molecular weight was determined through gel permeation chromatography (GPC) and DSC was used to determine the glass transition temperature of the PBMA polymer synthesized in confinement and bulk. Finally, scanning electron microscopy (SEM) has been performed to determine the final structure of the synthetized polymer nanostructures.

## 2. Materials and Methods

### 2.1. Materials

The monomers, butyl methacrylate (BMA) and 2-hydroxyethyl acrylate (HEA) and the initiator, 2,2′-azobis-(isobutyronitrile) (AIBN), were purchased from Sigma-Aldrich, Madrid, Spain. Monomethyl ether hydroquinone inhibitor present in BMA and HEA was removed by a silica gel column, and the initiator was purified by recrystallization from methanol before use.

Ultrapure aluminum foils (99.999%) from Advent Research Material were cleaned and degreased by sonication into solvents of different polarity (acetone, water and ethanol). Subsequently, an electropolishing process was carried out for 4 min in a solution of perchloric acid (70%)/ethanol (99.9%) (volume ratio 1:4) with constant voltage 20 V at temperature below 10 °C, in order to eliminate irregularities on the aluminum surface.

### 2.2. AAO Templates Used as Nanoreactors

Ordered pore structure of AAO templates of different pore diameters were prepared by a two-step electrochemical anodization process using aluminum foils, based on a Masuda et al. procedure [38] and developed in our laboratory [1,39]. Well-defined pore diameter and length of AAO nanopores were adjusted by controlling the anodization conditions (voltage, temperature, time and two types of electrolytes). Each electrolyte is suitable for a particular AAO pore diameter (narrow or wide) while AAO template length (short or long pore length) is adjusted as a function of time (see Table 1 for experimental conditions).

In order to obtain AAO templates of 60, 200, 350 and 400 nm of pore diameter, templates with initial pore diameter of 35 and 140 nm were widened in a 5 wt% phosphoric acid solution at 35 °C.

For AAO templates used in DSC polymerization, the aluminum layer of templates was removed by means of a partial solution in HCl, CuCl_2_ and H_2_O. After that, AAO templates were washed in H_2_O and dried.

### 2.3. In Situ Polymerization of BMA in AAO Nanoreactors

Free radical polymerization (FRP) of BMA was carried out into the nanocavities of AAO templates of 35, 60 and 300 nm of pore diameters with 1 and 100 µm of pore length. The polymerization reaction was followed by proton Nuclear Magnetic Resonance (^1^H-NMR) and Differential Scanning Calorimetry (DSC).

First, the “in situ” polymerization of BMA was studied by DSC by continuously monitoring the heat of exothermal reaction, as a function of time. For this purpose, a mixture of BMA and AIBN (0.5 wt%) was prepared in a flask under N_2_ in an iced bath and purged for 30 min. Then, the aluminum removed AAO template was introduced inside of monomer solution under vacuum for 30 min in order to fill the nanoreactors with the monomer. Afterwards, the AAO template was extracted from the flask, superficially cleaned and cut into small pieces to be stacked inside of a DSC aluminum hermetic pan of 50 μL, in order to achieve the highest amount of monomer to react. Finally, polymerization reactions were performed in isothermal conditions under nitrogen atmosphere at 50, 60 and 70 °C.

After the isothermal process, the system was cooled down to –30 °C and then heated to 150 °C at 10 °C/min in a dynamic process to check any residual heat of reaction and to determine the glass transition temperature (Tg) of the confined polymer.

The bulk polymerization of BMA monomer was also followed by DSC. To that aim, 10 mL of monomer solution were deposited into a DSC hermetic pan. The isothermal and dynamic processes were performed following the same method previously described at 50, 60 and 70 °C.

The study of in situ polymerization of BMA in AAO nanoreactors of 60 nm and 100 µm was also carried out by ^1^H-NMR spectroscopy as a function of time. This technique allows for the identification of the chemical structure of both BMA monomer and PBMA homopolymer and therefore, the study of polymerization kinetic. For this purpose, AAO nanoreactors were filled with the monomer solution (BMA and AIBN of 0.5 wt%) under vacuum. Next, the AAO template was removed from the solution, superficially cleaned, weighed and heated in an oven at constant temperature (60 °C) to start the polymerization reaction. At a certain reaction time, the AAO template was cooled in a freezer overnight to stop the reaction. Afterwards, the polymer synthetized within the AAO template was introduced in a vial with deuterated chloroform over 48 h to extract both the synthesized polymer and the residual monomer from the nanoreactors and their chemical structure was analyzed through ^1^H-NMR. This procedure was repeated at different reaction times to obtain conversion curves versus time.

Bulk polymerization of BMA was carried out in the same reaction conditions as previously described. In order to follow the polymerization kinetics, 0.5 mL of monomer mixture was purged with N_2_ and introduced in a ^1^H-NMR tube. A closed capillary filled with deuterated chloroform was introduced in the ^1^H-NMR tube in order to eliminate the influence of the solvent in the reaction. After that, spectra were registered at 60 °C every 3 min for 2.5 h.

### 2.4. Polymer Infiltration

Polymer infiltration process of PBMA in AAO templates was carried out via a melt precursor film method. Solid polymer material was placed on the AAO surface and then infiltrated at a temperature of 200 °C for 6 h [1].

### 2.5. Characterization Methods

A PerkinElmer DSC 8500 calorimeter with an intracooler was employed to monitor polymerization reaction under a nitrogen atmosphere and the glass transition temperature (Tg) of synthesized polymers, as described in the previous section.

Varian Inova 300 MHz and Bruker Avance III HD-400 MHz H-NMR spectrometers were used to monitor polymerization reaction as described in the previous section. Standard parameters used to acquire spectra: a pulse of 90° was employed with relaxation delay of 5 s. More than 32 single-scan ^1^H-NMR spectra were acquired in the majority of the kinetics experiments. Standard ^1^H-NMR tubes of 5 mm of diameter were used in the experiments. Experiments were carried out at 60 °C temperature.

Scanning electron microscopy (SEM) employing a FESEM Hitachi model SU8000 microscope was used for the morphological characterization of selected samples of AAO templates and polymer nanostructures. To perform the analysis of extracted free polymer nanopillars, the aluminum substrate of filled AAO samples was removed with a mixture of HCl, CuCl_2_, and H_2_O and the alumina was dissolved in 10% wt H_3_PO_4_. Previously, to support the free nanostructures, a coating was placed over the template [40].

Molecular weight analysis was carried out by Gel permeation chromatography (GPC) by means of Styragel Water columns (300 mm × 7.8 mm, 5 mm nominal particle size). THF was used as a solvent. Measurements were performed at 35 °C at a flow rate of 1 mL/min using an RI detector. Molecular weights of polymers were referenced to PS standards. The homopolymer synthesized in AAO nanoreactors was extracted from the nanocavities by submerging the template in a vial with THF and stirred for two days. Afterwards, it was placed in an ultrasound bath for several periods of 30 min. Then, the solution was filtered, precipitated in methanol and dissolved again in THF. In case of bulk homopolymer, sample was dissolved in THF and stirred during few hours.

Thermal stability characterization was carried out using TA Instruments TGA Q500. To conduct the TGA assay, the samples were subjected to a temperature ramp from 40 to 600 °C at 10 °C/min.

Contact angle (CA) measurements were carried out using a KSV Theta contact angle system. In a typical measurement, 0.75 μL droplet of water was deposited on the sample surface. The average contact value was obtained at five different positions of the same sample.

## 3. Results

**Figure 1:**
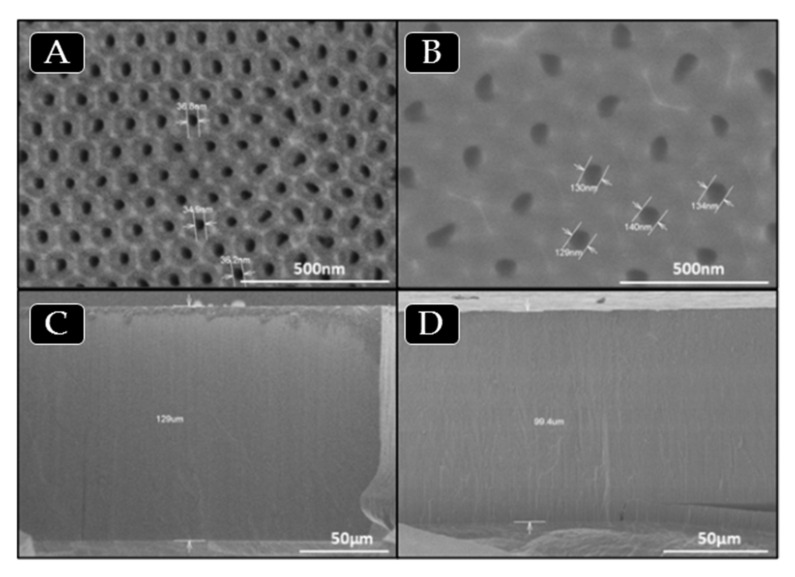
SEM images of AAO templates obtained with oxalic acid solution (**a**,**c**) and phosphoric acid (**b**,**d**) as electrolytes, respectively. Surface view of templates with 35 (**a**) and 140 (**b**) nanometers of pore diameter and cross section view of templates (**c**, **d**), where the length is around 100 µm, see values in Table 1.

The synthesis of AAO templates was carried out under different anodization conditions in order to obtain templates with nanopores of different sizes, as described in the Experimental section. AAO nanoreactors prepared by a two-step anodization process were characterized by SEM. Figure 1a–d show SEM images of AAO templates obtained by anodization with different electrolytes. Figure 1a,c show top view surface and cross section images of AAO templates obtained with 0.3 M oxalic acid solution as electrolyte, whereas, Figure 1b,d corresponds to AAO templates obtained with phosphoric acid 2 wt% as electrolyte.

### 3.1. Free Radical Polymerization of BMA in AAO Nanoreactors

#### 3.1.1. Kinetic Analysis by DSC

Firstly, the kinetics of the free radical polymerization reaction of the BMA monomer in confinement in AAO templates with different pore sizes were followed by DSC at different temperatures.

**Figure 2:**
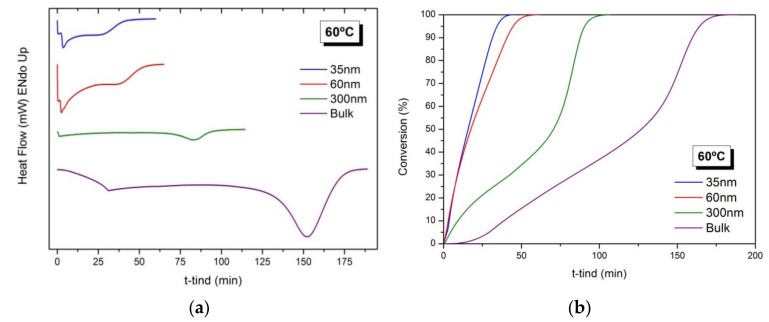
Free radical polymerization of BMA at 60 °C in confinement in AAO templates of 35, 60 and 300 nm of pore diameters and in bulk conditions. (**a**) Thermograms corresponding to the heat generated during the reactions and (**b**) Evolution of the conversion (%) as a function of time (min).

Figure 2a shows the heat generated during the polymerization reaction of BMA in confinement in AAO templates of 35, 60 and 300 nm and in bulk, under isothermal conditions at 60 °C. As observed in all thermograms, an exothermic peak appears that corresponds to the exothermic polymerization reaction. In addition, confinement conditions accelerate the polymerization process, yielding a narrower exothermic peak and, as the degree of confinement increases, with the decrease of the AAO pore size, the reaction rate increases. The results are in agreement with previous works for other acrylic monomers [16,17]. In these works, it was found that the decomposition rate of initiator increased in the presence of the alumina [25] and also with the degree of confinement [17].

The evolution of the conversions (*X*) of BMA homopolymerization as a function of time can be calculated from the heat generated during process by Equation (1) [25]:

(1) $$X=\frac{1}{∆H_{T}}\int_{t_{0}}^{t}Q\;dt\;$$

where Δ*H_T_* is the total heat of reaction and *Q* is the corresponding reaction heat flow.

Figure 2b depicts the evolution of the conversion (%) as a function of time for the polymerization reactions carried out in AAO templates of different pore diameters (35, 60 and 300 nm). For reactions in AAO templates of 300 nm and in bulk, a strong gel effect is observed, marked by the onset of a rapid polymerization at polymer conversions above 50%. For polymerization in AAO reactors of 35 and 60 nm, although not experimentally noticeable, it is assumed to be the same gel effect.

In addition, polymerization reactions at 50 and 70 °C under confinement conditions using AAO templates of 60 nm of pore diameter were also carried out. Figure S1a,b show the heat generated during the isothermal reactions and the evolution of the conversions as a function of time. As expected, as the reaction temperature increases, the reaction rate increases and autoacceleration also occurs at an earlier time. The polymerization reactions at 60 and 70 °C show an initial polymerization rate very high in both assays and very distant from the results obtained at 50 °C. We consider that the difference in both experiments is practically negligible within experimental errors.

Nevertheless, for the reaction carried at 50 °C, a residual heat was found, i.e., polymerization did not achieve 100% conversion. For this reason, a subsequent dynamic heating process was carried out in which a residual heat respect to the total heat of the reaction of 14% was noted (see Figure S2).

Table 2 summarizes the conditions and the analysis results of the reactions performed using Differential Scanning Calorimetry (DSC). From the data of the table, some general conclusions can be drawn.

First, under confinement conditions, the free radical homopolymerization reaction of BMA starts earlier than in bulk (see *t_ind_* at Table 2), in agreement with other results in the literature [16,25]. Second, the total reaction time (see *t_tot_*) decreases as the degree of confinement increases. Third, the average reaction enthalpy (Δ*H*) for BMA polymerization in confinement is 165 ± 16 J/g, similar to the heat reported in the literature for other acrylate monomers [25,34]. The differences in value in the case of confinement are attributed to the methodology of the experiment, where a weighing error of ±0.5 mg leads to values of ±100 J/g. Fourth, for the same degree of confinement at 60 nm, the higher the reaction temperature, the shorter the reaction time (*t_tot_*).

#### 3.1.2. Kinetic Analysis by ^1^H-NMR

In order to unambiguously assess the chemical structure of the synthesized polymer as well as to determine the kinetics reaction, the study of free radical polymerization of BMA in nanoreactors using AAO templates and bulk conditions was also followed by ^1^H-NMR spectroscopy.

First, the polymerization reaction was carried out in bulk at 60 °C (see experimental section). The ^1^H-NMR spectrum of a mixture of polymer and monomer was taken after 5 min of reaction, see Figure S3 of Supplementary Materials. From this spectrum, all the chemical shifts and proton signals has been assigned to the different chemical structures [41] of BMA monomer and PBMA and collected in Table S1 of Supplementary Materials.

**Figure 3:**
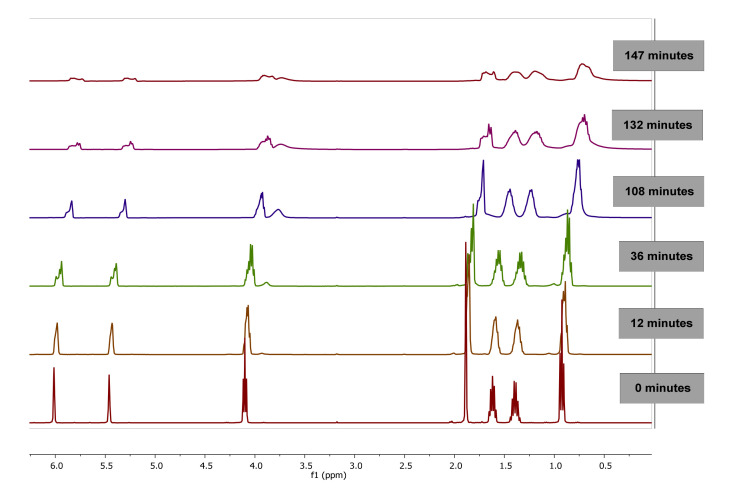
^1^H-NMR Spectra of poly(butyl methacrylate) (PBMA) obtained by bulk polymerization at 60 °C at different reaction time, using CDCl_3_-d.

The evolution of ^1^H-NMR spectra of polymerization of BMA in bulk at 0, 12, 36, 108, 132 and 147 min is plotted in Figure 3. As observed, as the reaction progresses, the value of integral corresponding to signals of double bond of vinyl protons (6.07 and 5.65 ppm) of BMA decrease due to monomer consumption while the integral of the proton signals between 0.5 and 2.0 ppm are broadened due to the polymer formation.

**Figure 4:**
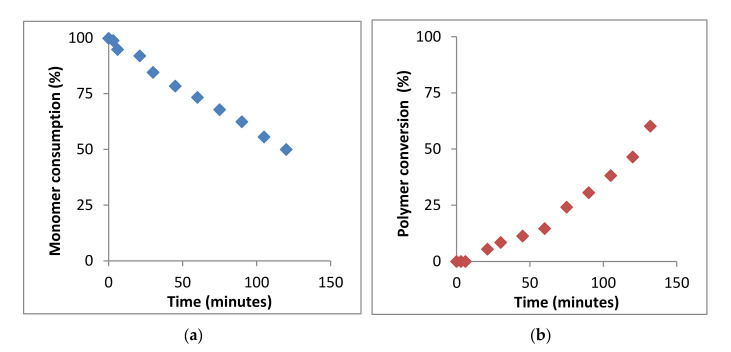
Kinetics of bulk polymerization of BMA at 60 °C, determined by ^1^H-NMR spectroscopy. (**a**) Monomer consumption and (**b**) polymer formation.

Therefore, the kinetics of the BMA homopolymerization can be studied from the monomer consumption and/or polymer conversion as a function of time by following the variation of the integrals value corresponding to signals of the vinyl protons at 5.55 and 6.07 ppm of BMA monomer and signals of 0.5 to 2.0 ppm of PBMA polymer, respectively (see Figure 4a,b).

As observed, in the polymerization process, until 147 min, the monomer consumption decreases moderately and the polymer conversion increases also moderately. Then, the kinetic plot was interrupted since ^1^H-NMR spectra offered very low resolution due to the high viscosity of the mixture and could not be further registered. The analysis of the BMA monomer consumption plot yields a reaction conversion of ~50% after 2.5 h, whereas the analysis of the signals corresponding to the PBMA yields a reaction conversion of ~60%. The results can be considered very similar, the difference being due to the low resolution of the ^1^H-NMR spectra.

**Figure 5:**
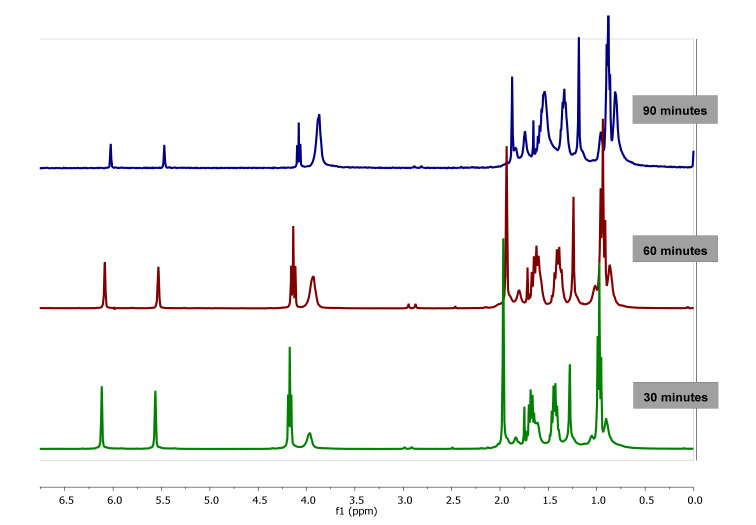
^1^H-NMR spectra of PBMA obtained by polymerization in confinement (nanoreactor diameter of 60 nm) at 60 °C, at different reaction time, using CDCl_3_-d.

The kinetics of polymerization of BMA in confinement in AAO templates of 60 nm pore diameter carried out at 60 °C was also studied by ^1^H-NMR spectroscopy by following the polymer conversion and monomer consumption, see experimental part for further details. As an example, the spectra taken after 30, 60 and 90 min of reaction time are plotted in Figure 5. As observed, when compared to bulk reaction, the spectra in confinement reaction show higher resolution than the spectra shown in Figure 3, probably due to the lower viscosity of the solution (attributed to the lower molecular weight of the polymer obtained, as it will be detailed in the following section) thus allowing the analysis of the kinetics of polymerization up to 100% conversion.

**Figure 6:**
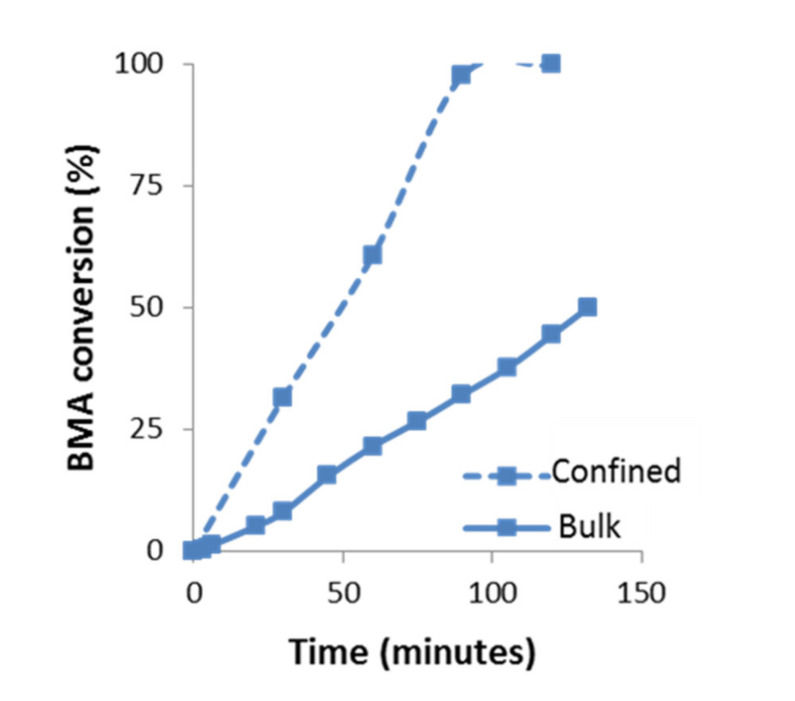
Kinetics of BMA polymerization at 60 °C under bulk and confinement conditions (AAO template of 60 nm), determined by ^1^H-NMR spectroscopy, using CDCl_3_-d.

The evolution of the BMA polymerization as a function of time in confinement conditions compared to bulk is plotted in Figure 6. Under confinement conditions, it is observed an increase in the reaction rate compared to bulk. For instance, after 1 h of reaction, the polymerization in confinement reaches ~60% of conversion while in bulk it only reaches ~22%. In confinement, the conversion trend is slightly self-accelerated until 90 min, where it has already reached 97% conversion.

An important consequence of these results is that by ^1^H-NMR spectroscopy in a confinement polymerization, it is possible to unambiguously determine the polymer formation as a function of time along the whole range of conversion (until 100%) by following the integral value of signals of BMA monomer consumption (vinyl protons at 5.55 and 6.07 ppm). This ^1^H-NMR monitoring methodology was validated to analyze the polymer formation of 2-hydroxyethyl acrylate monomer (HEA) in AAO templates by free radical polymerization in confinement (see Figure S4 of Supplementary Materials and corresponding results). As observed, the integral value of HEA monomer signals decrease as a function of time while integral of PHEA signals increases, so their evolution can be followed as a function of time. Moreover, as HEA signals are shifted respect to BMA signal, both signals can be independently analyzed. Therefore, the ^1^H-NMR method presents new possibilities to study the copolymerization reaction in confinement by ^1^H-NMR kinetic analysis, including the determination of chemical composition of the copolymer. In fact, this methodology has been successfully applied to study the copolymerization in confinement of butyl methacrylate and 2-hydroxyethyl acrylate and to determine the reactivity ratios of BMA and HEA monomers by atom transfer radical polymerization (ATRP) [42].

**Figure 7:**
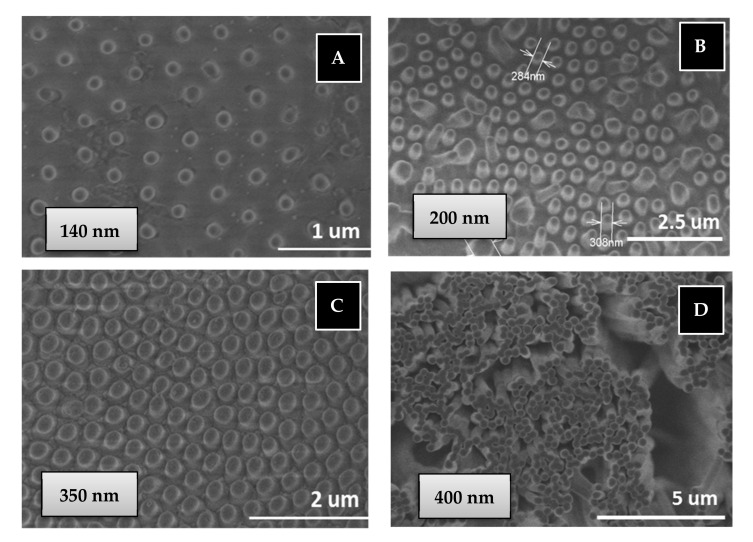
SEM images of PBMA nanopillars obtained by free radical polymerization (**B**) or infiltration (**A**,**C**,**D**) in AAO templates: (**A**) 140 nm diameter and 1 µm length; (**B**) 200 nm diameter and 2 µm length; (**C**) 350 nm diameter and 1 µm length; (**D**) 400 nm diameter and 100 µm length.

Another important consequence is that a 100% of PBMA nanostructures are obtained from the BMA monomer by the free radical polymerization in AAO templates through a quick double process: 30 min of monomer immersion under vacuum at room temperature and 90 min of polymerization reaction at 60 °C, while the same polymer nanostructures are obtained by polymer infiltration of PBMA in AAO templates at 200 °C over 6 h (see Figure 7). Therefore, the in situ polymerization method is a softer and quicker process with less energy consumption than traditional polymer infiltration method to obtain polymer nanostructures.

#### 3.1.3. Polymer Nanostructures

The number average molecular weight (Mn) of the nanostructured PBMA polymer obtained by polymerization in confinement (60–300 nm) is between 30 × 10^3^–175 × 10^3^ g/mol, whereas Mn for bulk polymer is 438·10^3^ g/mol. This trend is the same as that observed in previous studies on the radical polymerization of PMMA and PS, where we found a decrease in the molecular weight and a decrease of the polydispersity of the synthesized polymer under confinement conditions [23,25]. According to theoretical predictions [43], when PDI value is close to two, the termination mode would be by chain transfer or disproportionation. For PDI values much lower than two, the principal mode of termination would be by combination. Therefore, the results suggest that in our case, chain transfer or disproportionation is the main termination process of polymerization of BMA in bulk, while combination is predominant in confinement. Furthermore, the molecular weight of the PBMA obtained by FRP in bulk and in confinement is within the average value of other methacrylate polymers synthesized by FRP under similar conditions [25]. The molecular weight of the PBMA homopolymer obtained under confinement conditions is significantly reduced with respect to that obtained by bulk polymerization, nevertheless, it is high enough to maintain the polymer properties.

The glass transition temperature (*T*_g_) of the PBMA synthesized in confinement (400 nm) and bulk conditions determined by DSC is 38 and 31 °C, respectively. This trend in the results is similar to that found in the literature for PMMA synthesized by FRP mechanism [25,44].Moreover, our *T*_g_ values are similar to those observed by Sha et al. for PBMA infiltrated in AAO templates [7]. There, the increase in the Tg value was explained due to restriction of space of AAO geometry and the reduction of the mobility of polymer chains along the wall of the alumina nanopores, as a consequence of hydrogen interactions between the ester groups of PMMA and the hydroxyl groups of AAO surface [45]. Therefore, the increase in the glass transition temperature of PBMA polymer nanostructures obtained by polymerization of BMA in confinement using AAO nanoreactors is in agreement with a general *T*_g_ behavior according to the literature [16,17,23,25,44,46,47,48,49].

Regarding thermal stability of the nanostructure PBMA based on our own previous studies, we assumed that confinement does not appreciably change the thermal stability of bulk synthetized polymer [50], although we know this statement is still controversial [51,52]. The thermal decomposition observed of bulk synthesized PBMA is above 275 °C [53,54]. So, the nanostructured polymer is available for different common bio-applications in which no high stability is required. The morphological characterization of surface-supported nanostructured BMA homopolymer has been carried out using scanning electron microscopy (SEM) for two types of PBMA nanostructures of higher and lower aspect ratio, L/D: 250 (400 nm, 100 µm) and 2.8 (350 nm, 1 µm), respectively, obtained by “in situ” polymerization or polymer infiltration in AAO nanocavities, see experimental part. Figure 7 show top view of SEM images of supported nanostructured PBMA. Images A, B and C correspond to low aspect ratio PBMA nanopillars obtained in AAO templates of pore diameter of 140, 200 and 350 nm, respectively, and 1–2 micron of pore length. Image D corresponds to high aspect ratio PBMA nanopillars obtained in AAO templates of pore diameter of 400 nm and 100 micron of pore length.

As observed in Figure 7A–C, the nanostructured polymer surfaces are composed of ordered, straight and stiff nanopillars. The dimensions of nanopillars obtained are 140, 270 and 300 nm, being of similar dimensions to those of the AAO geometries. Figure 7D also shows homogenous, straight and stiff nanopillars/fibers of high aspect ratio. An important advantage is that nanostructured polymer can cover large surface areas of several cm^2^ (the same surface as that of AAO geometries).

PBMA nanostructures of 350 nm of diameter and 1 µm of length, in aqueous medium under swollen conditions, shows a Gaussian distribution of the Young’s modulus, with a value of 3.1 ± 1.1 MPa [42,55]. Nanopillars present low elastic value and it can be explained in terms of thermosensitive behavior of the polymer. According to the literature, this polymer is especially sensitive to temperature, being room temperature a key point at which the elastic-plastic properties of the polymer change abruptly [42,55,56,57,58]. In fact, as previously observed by us and reported in literature, the Tg lies within the range of 20–30 °C, so that at room temperature, the polymer is just in the transition interval to a soft material. This could explain its low elastic moduli and the elastic behaviour found.

The synthesis of polymer nanostructures by in situ polymerization process in AAO templates allows the tailoring of surface morphology as a function of AAO dimensions and, therefore, to modulate the polymer surface properties like adhesion, hydrophobicity and to provide basic guidance for biocompatibility studies of the material. The results of the contact angle measurement for the bulk BMA polymer show a hydrophobic character of the material (93 ± 3°). Then, PBMA homopolymer nanostructured combined with a hydrophilic polymer like 2-hydroxyethyl acrylate would form a substantially complementary material [42,55].

As a whole, PBMA polymer nanostructures easily obtained through free radical polymerization of BMA under AAO geometries, deliver a good balance of properties, that is, moderate Mn and thermal stability and tailored nano/micro/macro dimensions. The hydrophobicity, morphology, Young’s modulus, thermal stability, among others, provides a basic orientation for the choice of possible applications in relation to these properties, such as biocompatibility studies of the material for biomedical devices.

## 4. Conclusions

AAO geometries are suitable nanoreactors to prepare PBMA nanostructures of suitable size by free radical polymerization of BMA monomer. In fact, PBMA nanostructures of high and low aspect ratios have been successfully obtained through a quick double process: 30 min of monomer immersion at room temperature and 90 min of polymerization reaction at 60 °C.

The synthesis of BMA homopolymer using the FRP mechanism carried out inside of AAO nanoreactors showed significant differences on reaction kinetics with respect to that determined for the synthesis in bulk due to the effect of confinement. DSC and ^1^H-NMR analyses confirmed that the rate of polymerization under confined conditions is higher that when polymerization occurs in bulk. In addition, with the increase in the degree of confinement, a self-acceleration of the reaction was observed, which is much more noticeable when the polymerization takes place in AAO templates of pore size of 300 nm and in the bulk. A gel effect was observed in all cases. Compared to the polymerization of BMA in the bulk, no limiting conversion was observed for the homopolymerization of BMA in confinement, and the gel-effect was observed at shorter times with the increase of temperature.

Moreover, the comparison of the results obtained through DSC and ^1^H-NMR for radical polymerization in AAO nanoreactors and in the bulk, allows to demonstrate that both techniques show similar trends and, therefore, they are both valid for the kinetic study of the polymerization reaction in confinement. The first has the advantage of the speed of the procedure, as long as it has the support of the second from which the formation of the polymeric structure can be ascertained. Furthermore, the ^1^H-NMR technique has been consolidated as a tool for studying the kinetics of the homopolymerization and copolymerization reactions in confinement.

PBMA is a hydrophobic polymer with the Young’s modulus dependent on the working temperature. PBMA obtained by radical polymerization under confined conditions has a moderately high molecular weight, as it is less polydisperse than that obtained in the bulk. It also presents a higher Tg than that obtained for bulk PBMA, due to the restrictions on mobility produced by the confinement. For PBMA obtained by radical polymerization under confined conditions, it is assumed a typical thermal stability of acrylates associated to polymer depolymerization.

In conclusion, homopolymerization of BMA in confinement using AAO pores as nanoreactors is a direct, fast and less energetic process than a polymer infiltration method. The homopolymer obtained presents properties that makes it suitable for applications in nanotechnology, either alone or in combination with other polymers (through copolymerization).

## Figures and Tables

**Table 1:** Anodization conditions.

AAO Pore Diameter (nm)	Electrolyte	Voltage (V)	Electrolyte Temperature (°C)	First Reaction Time (h)	Second Reaction Time	AAO Pore Length (µm)
35	0.3 M H_2_C_2_O_4_	40	1–2	24	30 min	1
					72 h	140
140	2 wt% H_3_PO_4_; 0.02 M C_6_Al_2_O_12_	195	0.5–1	6	15 min	1
					24 h	100

**Table 2:** Kinetic parameters of BMA free radical polymerization in confinement and bulk conditions at different temperatures determined, obtained by DSC.

Pore Diameter AAO (nm)	Temp. (°C)	$t_{ind}\;(\min)$	$t_{tot}\;(\min)$	ΔH (J/g)
35	50	*	97	161
	60	*	46	137
60	50	*	145	163
	60	*	57	174
	70	*	48	177
300	60	12	107	179
Bulk	50	158	343	341
	60	22	189	414
	70	8	94	392

* Values in the range of seconds.

## Data Availability

Not applicable.

## Supplementary Materials

The following are available online at https://www.mdpi.com/2073-4360/13/4/602/s1, Figure S1: Free radical polymerization of BMA at 50, 60 and 70 °C in confinement in AAO templates of 60 nm of pore diameters and in bulk conditions. (a) Thermograms corresponding to the heat generated during the reactions and (b) Evolution of the conversions (%) as a function of time (min); Figure S2: Residual heat from dynamic process applied after isothermal polymerization reaction of BMA in 60 nm AAO nanoreactors at 50 °C; Figure S3. 1H-NMR Spectrum of BMA bulk polymerization at 60 °C after 5 min of reaction time using CDCl3-d; Table S1: 1H-NMR Signal Assignments of BMA monomer and PBMA homopolymer; Figure S4: Results of monitoring bulk and confined (60 nm diameter nanoreactor) free radical polymerization of 2-hydroxyethyl acrylate (HEA) at 60 °C by 1H-NMR. (a) 1H-NMR Spectra of PHEA obtained by polymerization in confinement, at different reaction time, using CDCl3-d; (b) Conversion (%) and reaction time (min) obtained in confined and bulk homopolymerization.
