# Supplementary material for: In Situ Synthesis of Poly(butyl methacrylate) in Anodic Aluminum Oxide Nanoreactors by Radical Polymerization: A Comparative Kinetics Analysis by Differential Scanning Calorimetry and 1H-NMR
Source: Polymers (Basel). 2021 Feb 17;13(4):602. doi: 10.3390/polym13040602 (PMC7923008; doi:10.3390/polym13040602)
Supplement: Supplementary file 1 [file polymers-13-00602-s001.pdf]

polymers

Supplementary Materials: In Situ Synthesis of Poly(butyl Methacrylate) in Anodic Aluminum Oxide Nanoreactors by Radical Polymerization: A Comparative Kinetics Analysis by Differential Scanning Calorimetry and ^{1}H-NMR

Laia León-Boigues, Luis Andrés Pérez and Carmen Mijangos

Figure S1a show the heat generated during the isothermal reactions of Free radical polymerization of BMA at 50, 60 and 70 °C under confinement conditions using AAO templates of 60 nm of pore diameter. Figure S1b show the evolution of the conversions as a function of time. As expected, as the reaction temperature increases, the reaction rate increases and autoacceleration also occurs at earlier time.

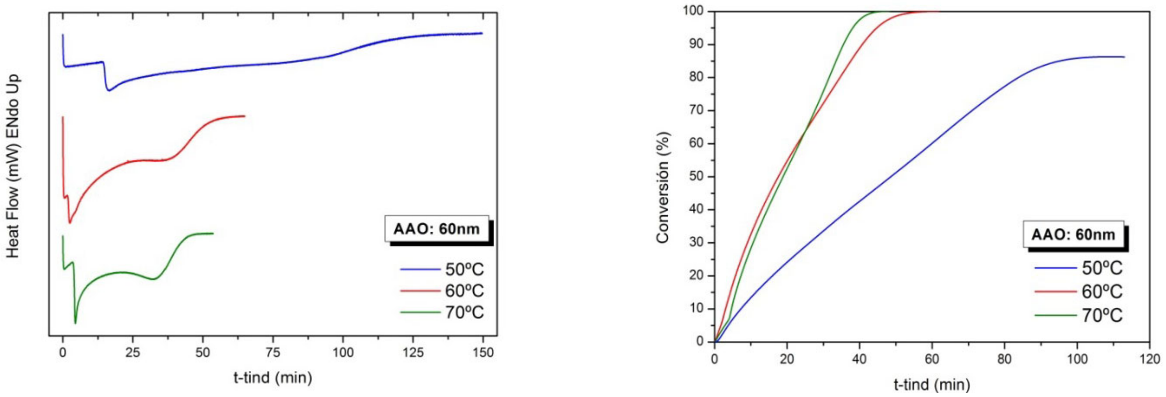

(**a**) (**b**)

Figure S1. Free radical polymerization of BMA at 50, 60 and 70 °C in confinement in AAO templates of 60 nm of pore diameters. (a) Thermograms corresponding to the heat generated during the reactions and (b) Evolution of the conversions (%) as a function of time (min).

Figure S2 shows the dynamic heating process carried out after the isothermal reaction of Free radical polymerization of BMA at 50 °C. Polymerization reaction did not achieve 100 % conversion and a residual heat was found.

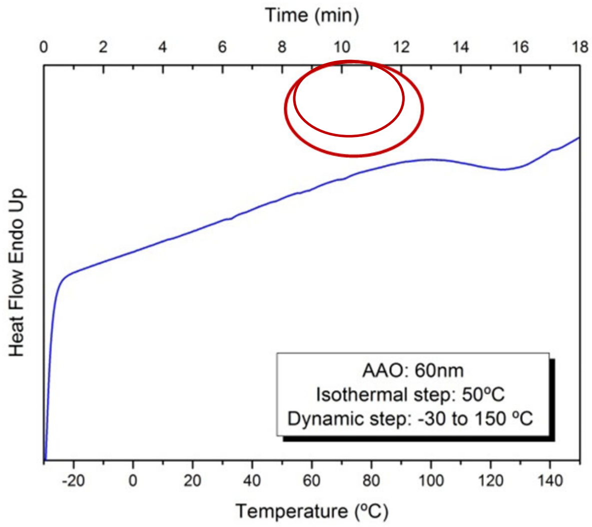

Figure S2. Residual heat from dynamic process applied after isothermal polymerization reaction of BMA in 60 nm AAO nanoreactors at 50 °C.

Figure S3. show the ^{1}H-NMR spectrum after 5 minutes of the polymerization reaction of BMA in bulk at 60 °C, showing a mixture of polymer and monomer. All the chemical shifts and proton signals has been assigned to the different chemical structures of BMA monomer and PBMA and collected in Table S1.

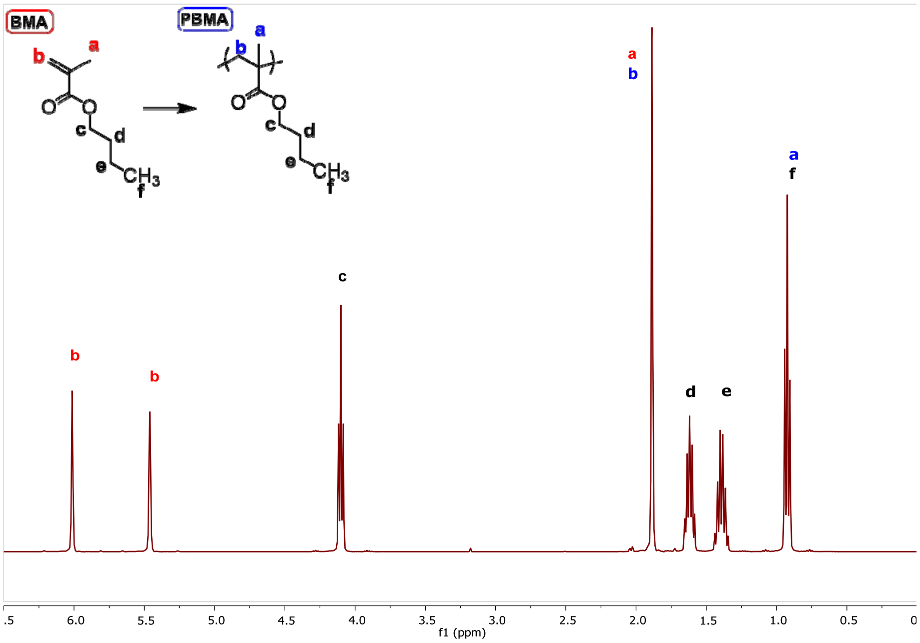

Figure S3. ^{1}H-NMR Spectrum of BMA bulk polymerization at 60 °C after 5 minutes of reaction time using $CDCl_3$-d.

Table S1. ^{1}H-NMR Signal Assignments of BMA monomer and PBMA homopolymer.

Assignation	Displacement (ppm)		Proton type
	Monomer	Polymer	
**a**	1.94	0.95	CH_2=C-(CH_3) → -CH_2-C-(CH_3); Methyl group
**b**	5.55<br>6.07	1.94	CH_2=C-(CH_3) → -CH_2-C-(CH_3); Vinyl groups
**c**	4.14		COO-CH_2- ; Methylene / Ester group
**d**	1.20-1.85		-CH_2- ; Methylene group
**e**	1.20-1.85		-CH_2- ; Methylene group
**f**	0.95		CH_3- ; Methyl group

The methodology of monitoring confined free radical polymerization by ^{1}H-NMR was applied to analyze the 2-hydroxyethyl acrylate (HEA) polymer formation in AAO templates. The experimental procedure has been the same as described in Materials and Methods to analyze BMA polymerization.

Figure S4. show the results of monitoring bulk and confined (60 nm diameter nanoreactor) free radical polymerization of 2-hydroxyethyl acrylate (HEA) at 60 °C by ^{1}H-NMR. (a) ^{1}H-NMR Spectra of PHEA obtained by polymerization in confinement, at different reaction time, using $CDCl_3$-d; (b) Conversion (%) and reaction time (min) obtained in confined and bulk polymerization.

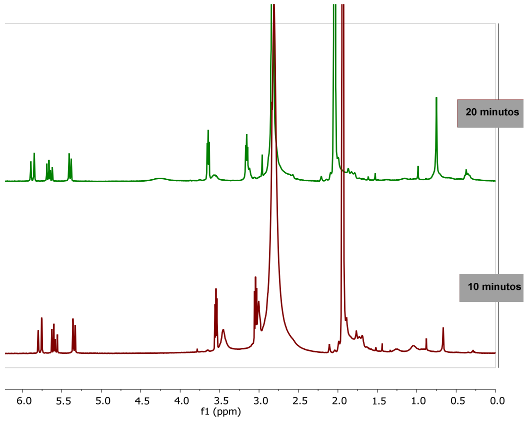

(a)

Confined (60 nm)		Bulk	
Reaction time (min)	Conversion polymer (%)	Reaction time (min)	Conversion polymer (%)
0	0	0	0
10	51.35	8	11.74
15	64.39	13	62.51
20	71.66	20	79.42

(b)

Figure S4. Results of monitoring bulk and confined (60 nm diameter nanoreactor) FRP polymerization of 2-hydroxyethyl acrylate (HEA) at 60 °C by ^{1}H-NMR. (a) ^{1}H-NMR Spectra of PHEA obtained by polymerization in confinement, at different reaction time, using $CDCl_3$-d; (b) Conversion (%) and reaction time (min) obtained in confined and bulk polymerization.
